# Clinical effectiveness and micro-perfusion alteration of Jingui external lotion in patients with knee osteoarthritis: study protocol for a randomized controlled trial

**DOI:** 10.1186/s13063-015-0661-x

**Published:** 2015-03-28

**Authors:** Da Guo, Xue-Wei Cao, Jin-Wen Liu, Wei Niu, Zhen-Wei Ma, Ding-Kun Lin, Jia-Yi Chen, Wei-Dong Lian, Wen-Wei Ouyang, Jun Liu

**Affiliations:** Department of Orthopedic Surgery, The Second School of Clinic Medicine, Guangzhou University of Chinese Medicine, No. 111 Dade Road, Guangzhou, Guangdong 510120 China; Department of Orthopedic Surgery, Hospital of Traditional Chinese Medicine of Zhongshan, No. 3 Xunkang Road, Zhongshan, Guangdong 528400 China; Department of Orthopedic Surgery, Hospital of Traditional Chinese Medicine of Meizhou, No. 35 Meishong Road, Meizhou, Guangdong 514000 China; Department of Statistical Secretary, The Second School of Clinic Medicine, Guangzhou University of Chinese Medicine, No. 111 Dade Road, Guangzhou, Guangdong 510120 China

**Keywords:** Knee osteoarthritis, Jingui external lotion, Clinical effectiveness, Micro-perfusion alteration, Dynamic contrast-enhanced MRI, Randomized controlled trial

## Abstract

**Background:**

Knee osteoarthritis is a major cause of disability in the aging population. Based on pathological, magnetic resonance imaging (MRI) and arthroscopy studies, progressive osteoarthritis involves all tissues of the joint and includes bone marrow lesions, synovial proliferation, fat pad inflammation, and high subchondral bone turnover. Recent research suggests that abnormal perfusion in bone marrow lesions, fat pads, and subchondral bone is associated with pain in knee osteoarthritis, and that dynamic contrast-enhanced MRI is a promising method for studying micro-perfusion alteration in knee osteoarthritis. Traditional Chinese Medicine approaches have been employed for thousands of years to relieve knee osteoarthritis pain. Among herbal medicines, the Jingui external lotion is the preferred and most commonly used method in China to reduce pain in patients with knee osteoarthritis; however, there is a lack of validated evidence for its effectiveness. The purpose of this study is to explore the effectiveness of Jingui external lotion for the management of painful knee osteoarthritis in a short-term study. In addition, we will assess micro-perfusion alteration in the patellar fat pad as well as the femur and tibia subchondral bone via dynamic contrast-enhanced MRI.

**Methods/design:**

This trial is a randomized, controlled study. A total of 168 patients will be randomized into the following two groups: 1) the Jingui external lotion group (treatment group); and 2) the placebo lotion group (control group). In both groups, lotion fumigation and external washing of the patients’ knees will be administered twice a day for 14 consecutive days. Follow-up will be at regular intervals during a 4-week period with a visual analog scale to assess pain, and additional characterization with the Western Ontario and McMaster Universities Index score; rescue medication will be recorded as the extent and time pattern. In addition, micro-perfusion alteration in the patellar fat pad, femur and tibia subchondral bone will be assessed via dynamic contrast-enhanced MRI.

**Discussion:**

This study will provide clinical evidence of the efficacy of Jingui external lotion in treating knee osteoarthritis, and it will be the first randomized controlled trial to investigate micro-perfusion alteration of knee osteoarthritis with Traditional Chinese Medicine external lotion via dynamic contrast-enhanced MRI.

**Trial registration:**

ClinicalTrials.gov identifier: ChiCTR-TRC-14004727; 31 May 2014.

## Background

Knee osteoarthritis (KOA) is a major cause of disability in the aging population and a burden on healthcare resources [1,2]. The risk of disability (defined as needing help walking or climbing stairs) attributable to KOA is as great as that attributable to cardiovascular disease and greater than that due to any other medical condition in elderly persons [3]. Pathologically, osteoarthritis (OA) is characterized by progressive loss of articular cartilage and new bone formation on conventional radiography. However, it is increasingly apparent, based on pathological, magnetic resonance imaging (MRI) and arthroscopy studies, that progressive OA involves all joint tissues including bone marrow lesions, synovial proliferation, fat pad inflammation, and high subchondral bone turnover [4-7]. This progressive joint failure causes pain, physical disability, and psychological distress, although many with structural and radiological changes consistent with OA are asymptomatic [8]. In recent years, more attention has been placed on micro-perfusion alteration in KOA. Studies [9-11] employed dynamic contrast-enhanced (DCE) MRI to investigate the association between knee pain and periarticular perfusion variables. These results suggest that abnormal perfusion including bone marrow lesions, fat pad, and subchondral bone as detected by DCE MRI are associated with pain in KOA and that DCE MRI is a promising method for studying micro-perfusion alteration in KOA.

The aim of KOA treatment is to reduce pain, improve physical function, prevent disability, and enhance the quality of life over the short term. Therefore, as aging and comorbidities increase, a more convenient approach is urgently needed. In China, and increasingly worldwide, pharmacological therapy guidelines recommend topical medications as an alternative, adjunctive therapy, or even first-line therapy for KOA [12,13]. Before oral administration, topical treatments are usually recommended to relieve mild or moderate KOA pain because of their favorable safety profiles. To relieve KOA pain, herbal external therapy, including plasters, ointments, and lotions, have been used under the principle of syndrome differentiation based on Traditional Chinese Medicine (TCM) theory for thousands of years. External plasters have been reported to be very important therapies that induce muscle relaxation and invigorate blood circulation [14,15]. Jingui external lotion with fumigation and washing therapy has been employed as an effective prescription to relieve KOA pain or joint stiffness over decades, but no randomized controlled trial evidence has been published. Published Chinese literature has documented that Jingui external lotion is efficacious in treating KOA; however, these interventions have not been rigorously evaluated, and have been regarded to be less credible due to a lack of objective evidence. Therefore, we designed a randomized, single-blind, placebo-controlled trial to explore the effectiveness of Jingui external lotion for the management of painful KOA in a short-term study and the micro-perfusion alteration in the patellar fat pad, femur and tibia subchondral bone via DCE MRI.

## Methods/design

### Study design

This is a randomized, double-blind, placebo-controlled trial. The trial protocol has been approved by the Research Ethical Committee of Guangdong Provincial Hospital of TCM, (reference B2014-001-01). The trial will be conducted in accordance with the Helsinki Declaration and will be monitored by the trial agency at Guangdong Provincial Hospital of TCM.

### Recruitment and consent

**Figure 1:**
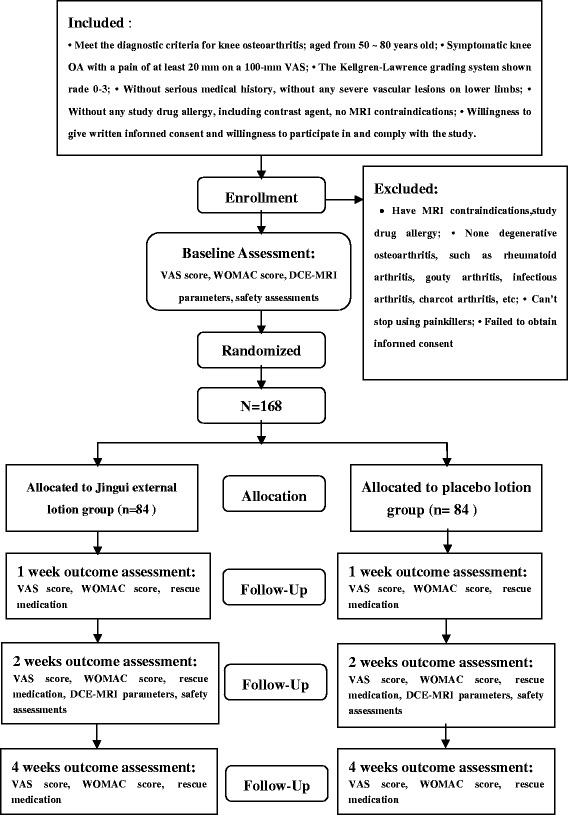
**Study flow chart.** DCE MRI, dynamic contrast-enhanced magnetic resonance imaging; OA, osteoarthritis; VAS, visual analogue scale; WOMAC, Western Ontario and McMaster Universities Index.

KOA patients scheduled for conservative treatment at the Outpatient Department of Orthopedic Surgery, Guang Dong Provincial Hospital of TCM, will be recruited, with a target sample size of 168 subjects. All candidates will go through a standardized interview process and receive more information about the study and the treatments. Written consents will be obtained. The purpose, procedures, and potential risks and benefits of the study will also be explained thoroughly to the participants. The more painful knee will be chosen as the study knee, and if both knees have the same symptoms we will choose the right side. The participants will be able to withdraw from the study at any time without consequence. The trial will be executed from March 2014 to March 2016 including enrollment and follow-up (Figure 1).

### Inclusion criteria

Participants meeting the following criteria will be included:

- Meet the diagnostic criteria for KOA (American College of Rheumatology criteria [16])
- Aged from 50 to 80 years old
- Symptomatic KOA with pain of at least 20 mm on a 100-mm visual analogue scale (VAS)
- Grade 0 to 3 on The Kellgren-Lawrence grading system
- No serious medical history or severe vascular lesions on lower limbs
- No known drug allergies, including to the contrast agent, and no MRI contraindications
- Willingness to give written informed consent and willingness to participate in and comply with the study.

### Exclusion criteria

Participants meeting one or more of the following criteria will be excluded:

- MRI contraindications, or study drug allergy
- Non-degenerative OA, such as rheumatoid arthritis, gouty arthritis, infectious arthritis, Charcot arthritis, and so forth
- Unable to discontinue use of painkillers
- Unwilling to give informed consent.

### Interventions

Eligible patients will be randomized into the following two groups: the Jingui external lotion group (treatment group) and the placebo lotion group (control group). The enrolled patients will be administered lotions via fumigating and washing externally for 2 weeks on both knees. DCE MRI examination will be performed at baseline and 2 weeks after the intervention is completed. The participants will be given personal instructions for using the lotions by our research nurses, who will be trained before the study. Patient visits will be performed at baseline and at 1, 2, and 4 weeks after treatment. Assessments including the VAS score for pain, Western Ontario and McMaster Universities Index (WOMAC) score and complications will be made during all four visits on the study knee.

The treatment group will receive Jingui external lotion, to be used twice a day (morning and evening, approximately every 12 hours and at the same time each day) for 14 consecutive days. The Jingui external lotion will be made according to the following procedure: a mixture of TCM (composed of *Semiliquidambar cathayensis* 60 g, *Radix zanthoxyli* 60 g, *Radix aconiti kusenzoffii* 30 g, *Radix aconite* 30 g, *Tinospora sinensis merr* 30 g, *Erythrina indica lam* 30 g, *Rheum officinale* 30 g, *Cassia twig* 30 g) will be boiled in 300 ml water, reduced to 150 ml of liquid medicine, and cooled to approximately 40°C. These hot liquid medicines will be administered as Jingui external lotion to the patients’ knees.

The control group will receive placebo lotion containing a food coloring agent and a small amount of Rheum officinale that is identical to the treatment group in terms of color and odor. The manufacturing and treating procedure are the same as the treatment group.

The research nurses will be involved in the treatment procedure to ensure the correct treatment is administered and that the temperature of the lotions is tolerable to the patients. During the process, when patients experience moderate pain or VAS score over 30 mm, ibuprofen sustained-release capsules will be administrated as rescue medication. Rescue medication consumption and time will be recorded in each randomized group when used. The patients will not be allowed to use other drugs aimed at treating KOA.

### Dynamic contrast-enhanced magnetic resonance imaging examination

The subjects will receive DCE MRI on the study knee at baseline and 2 weeks after intervention using a 3.0 T scanner (Magnetom Tim Trio; Siemens Medical Solutions, Erlangen, Germany) with an eight-channel transmit-receive phased array knee coil (In vivo Corporation, Gainesville, FL, USA). The knee imaging protocol will consist of a sagittal three-dimensional high-resolution T1-weighted fast low angle shot sequence with selective water excitation (TR/TE = 500/20 ms; flip angle = 25; FOV = 18.0 cm; slice thickness = 3 mm; matrix = 320 × 240; receiver bandwidth = 200 Hz/pixel) as well as a sagittal T2-weighted fat-saturated spin echo (TR/TE = 4790/84 ms; FOV = 18.0 cm; slice thickness = 3 mm; matrix = 320 × 240; receiver bandwidth = 130 Hz/pixel). The synovial membrane will be evaluated using DCE sagittal three-dimensional T1-weighted fast low angle shot sequence with the following parameters: TR/TE = 12/3.9 ms, flip angle = 60; FOV = 15 × 15 cm, slice thickness = 5 mm, matrix = 256 × 128, receiver bandwidth = 200 Hz/pixel, temporal resolution = 30 seconds. This sequence will be acquired in contiguous 5-mm sagittal slices throughout the knee before, during and after intravenous bolus administration of double-dose contrast agent gadolinium diethylene triamine pentaacetic acid (0.2 mg/kg). Baseline pre-contrast static images as well as DCE images will be acquired after bolus injection [17,18]. The total acquisition time for the imaging protocol is 24 minutes.

Three-dimensional images of the knee joint will be rebuilt using a Leonardo workstation (Siemens Medical Solutions INC USA, 2501 North Barrington Rd, Hoffman Estates Il 60195). The sagittal DCE sequence images and the intensity-time curve will be attained with the mean curve function. Four similar subchondral rectangular regions of interest (pixel area of 30) will be selected in the medial and lateral compartment of the distal femur and tibia plateau as well as the infra-patellar fat pad and supra-patellar fat pad. Measurement of these regions will be performed in terms of the enhanced rate and maximum upslope.

### Outcome measures

#### Primary outcome measure

The primary efficacy endpoint of the study will be VAS, which is a pain score ranging from 0 mm (no pain) to 100 mm (worst pain ever experienced) [19], measured during all the assessment visits (baseline and 1-, 2-, and 4-week follow-up). The VAS score is usually a horizontal line, 100 mm in length, anchored by word descriptors at each end. Patients mark the point of their current pain on the line. The VAS score is then determined by measuring in millimeters from the left end of the line to the point that the patient marked.

#### Secondary outcome measure

The secondary efficacy endpoint of the study will be rescue medication, WOMAC score [20] and micro-perfusion alteration in the patellar fat pad, femur and tibia subchondral bone.

Rescue medication complicates the interpretation of trial results by having an effect on the trial outcome. It is also possible to use rescue as a trial outcome. This is common in trials of treatments for asthma, where use of β_2_-agonists for rescue therapy may be a secondary trial outcome [21]. Rescue medication in this trial is Ibuprofen, which is a short-term nonsteroidal anti-inflammatory drug. We assume that the use of rescue medication varies with time in subjects in response to worsening of their disease. Consumption and time the subjects use rescue medication will be recorded in each randomized group.

The WOMAC score is a widely used proprietary set of standardized questionnaires used by health professionals to evaluate the condition of patients with osteoarthritis of the knee and hip, including pain, stiffness, and physical functioning of the joints. The index measures five items for pain (score range 0 to 20), two for stiffness (score range 0 to 8), and 17 for physical function (score range 0 to 68). It will be measured during all the assessment visits (baseline and 1-, 2-, and 4-week follow-up).

To assess micro-perfusion alteration, two parameters will be used: 1) maximum upslope represents the initial uptake of gadolinium into the region measured by finding the fastest uptake in the first few minutes after injection; 2) an enhanced rate represents the efflux rate constant from the extravascular extracellular space to plasma, which provides an index of venous hypertension and represents the ratio of the permeability surface area product over the extravascular extracellular space. With the mean curve function, maximum upslope and enhanced rate in the distal femur and tibia plateau subchondral regions of interest, and the infra-patellar fat pad and supra-patellar fat pad regions of interest will be measured and assessed.

### Safety assessments

All subjects will be questioned about adverse events during the treatment at each visit, and all adverse events reported will be analyzed, regardless of the investigators’ assessments of causality. Safety will be assessed by complete blood cell count, erythrocyte sedimentation rate, blood chemistry, and urinalysis.

### Randomization and blinding

Random assignment will be performed after consent is obtained using a computer-generated, blocked random-allocation sequence with a 1:1 ratio. The trial will be blinded to both patients and assessor. We will designate an independent researcher who is blinded to the interventions to be the assessor.

### Sample size

Calculation of sample size is based on previous studies assessing the efficacy and safety of Weishi Bitong Xifang fumigation for mild and moderate KOA in patients [22]. Group sample sizes of 73 and 73 achieve 90% power to detect a difference of 0.7 between the null hypothesis that both group means are 30 mm (VAS score uses a 100-mm linear measure) and the alternative hypothesis that the mean for the placebo group is 23 with estimated group standard deviations of 12 and 13 and with a significance level (alpha) of 0.05 using a two-sided test. The number of patients actually provides less than 90% power, assuming a withdrawal rate of 20%. Therefore, we will recruit a total of 168 patients; 84 patients in each group.

### Statistical analysis

The data will be collected and analyzed according to the intention-to-treat principle. We will compare baseline characteristics in both groups. Efficacy analyses will be performed for both the intent-to-treat population and per-protocol population. The intent-to-treat population will consist of all randomized subjects who have been administered at least one treatment. In a per-protocol analysis, only patients who complete the entire clinical trial according to the protocol are counted in the final results. Primary outcome will be compared between both groups. The extent and time pattern of rescue medication will be reported in each randomized group; when comparing the underlying outcomes of the rescue medication, we assume the use of rescue medication varies with time, to allow for dependence within patients, so we adopt a multilevel regression approach with rescue as a time-dependent covariate [23]. Micro-perfusion alteration outcome will be compared using Student’s *t*-test. All statistical analyses will be performed using SAS 9.2 software (SAS Institute Inc, Cary, NC, USA). All statistical tests will be two-sided, and the level of significance will be set at 0.05.

## Discussion

This study will provide clinical evidence on the efficacy of Jingui external lotion in treating KOA, and it will be the first randomized controlled trial to investigate micro-perfusion alteration of KOA with TCM external lotion using a DCE MRI technique. In TCM, KOA development is attributed to deficiency of the liver and kidney, and deficiency of qi and blood, attack of wind-cold-dampness evils, obstruction of channels and collaterals, qi and blood stagnation [24]. Thus, TCM that can promote blood circulation, remove blood stasis, and activate meridians to stop pain may be effective for KOA [13]. Fumigation and washing of TCM has recently been considered an inexpensive and convenient therapeutic approach for KOA [25]. Jingui external lotion is a classical and well-known external therapy in the orthopedic departments of TCM and has been demonstrated to provide a satisfactory effect for KOA in the clinic. Well-designed randomized controlled trials are needed to examine the efficacy of TCM treatments for OA.

It has been revealed that Chinese medicine promoting circulation and removing stasis could reduce intraosseous pressure [26]. Intraosseous hypertension has been reported to have an association with deep pain in patients with KOA [27]. To date, there is a lack of evidence to support the hypothesis that Chinese medicine promotes circulation; therefore, we will employ a DCE MRI technique to study micro-perfusion alteration, with the aim of attaining objective evidence of Jingui external lotion effectiveness in the treatment of patients with KOA.

## Trial status

Recruitment commenced in March 2014, and the trial is scheduled to end in March 2016.

## Abbreviations

DCE: dynamic contrast-enhanced
KOA: knee osteoarthritis
MRI: magnetic resonance imaging
OA: osteoarthritis
TCM: Traditional Chinese Medicine
VAS: visual analogue scale
WOMAC: Western Ontario and McMaster Universities Index
